# Young Children Feeding Practices: An Update from the Sultanate of Oman

**DOI:** 10.3390/children8090818

**Published:** 2021-09-17

**Authors:** Salima Al Maamari, Saleh Al Shammakhi, Ibtisam Alghamari, Jana Jabbour, Ayoub Al-Jawaldeh

**Affiliations:** 1 Nutrition Department, Ministry of Health, Muscat 393, Oman; dr.salima.almamary@gmail.com (S.A.M.); Saleh9959@gmail.com (S.A.S.); ibtisam.alghammari@moh.gov.om (I.A.); 2 Nutrition Department, School of Health Sciences, Modern University of Business and Sciences, Beirut 113-7501, Lebanon; 3 Regional Office for the Eastern Mediterranean (EMRO), World Health Organization (WHO), Cairo 11371, Egypt; aljawaldeha@who.int

**Keywords:** breastfeeding, diet diversity, acceptable diet, infant, children under two

## Abstract

Despite proven benefits, most countries fail to meet international targets for appropriate complementary and Breast Feeding (BF) practices. This study assessed feeding practices of children under two years of age and correlated them with family parameters in Oman, a high income country in the Eastern Mediterranean Region. Methods: Data from this study originated from the latest Oman National Nutrition Survey (ONNS). Assessment of children and their mothers’ socioeconomic, anthropometric, and nutritional variables was conducted at the household level. Evaluated feeding practices included age appropriate BF, diet diversity, and minimum acceptable diet (MAD). Results: Pairs of mothers and infants (n = 1344) were assessed. Early BF, exclusive BF at 6 months, infant formula, and iron rich meals were provided to 81, 29, 44, and 84% of children, respectively. Age appropriate BF and MAD were found in 58% and 35% of children, respectively. Low maternal education, younger age, low household income, and governorate negatively affected diet acceptability. Conclusion: Omani children successfully received early BF postpartum and consumed iron rich meals. Yet, rates of exclusive BF rates at six months and MAD for children under two were low. Comprehensive strategies should be placed to assess and influence children feeding practices in the Sultanate.

## 1. Introduction

The first 1000 days of life, lying between conception and the child’s second birthday, present a window of opportunity where nutrition and lifestyle changes mold the child’s development and his/her risk of developing chronic diseases in adulthood [1]. In this time window, high diet quality and age appropriate Breastfeeding (BF) affect infant growth and development as well as the risk of developing Non Communicable Diseases (NCDs). BF is associated with decreased risks of ear and gastrointestinal infections in infancy, a more diverse gut microbiota, and a reduced incidence of obesity and NCDs later in life [1,2]. BF positively influences mothers who benefit from improved emotional bonds with their offspring, and reduced incidence of type 2 diabetes, ovarian and breast cancer. At the community level, BF is associated with reduced costs and contributes towards a sustainable diet [3]. Along with BF, a nutritious diet is of key importance for optimal growth. High diet quality assessed by diversity and frequency of meals among children under two years of age was associated with satisfactory infant growth and development [4,5,6,7]. In light of these benefits, and in line with the Sustainable Development Goals, the World Health Organization (WHO) and the United Nations International Children’s Emergency Fund (UNICEF) identified Global Nutrition Targets for exclusive BF of 50% by 2025 and of 70% by 2030 [8]. WHO recommends early initiation of BF, exclusive provision of breastmilk for the first six months of life, continued BF until two years of age, and a diverse diet with appropriate meal frequency [9,10]. Yet, despite the awareness of the importance of these recommendations, they remain poorly followed, with less than half of infants around the world being breastfed, and less than a third of children having a diverse diet [11,12,13,14].

The Sultanate of Oman, a member country of the Gulf Cooperation Council, is a high income country, located in the Eastern Mediterranean Region (EMR). With the discovery of oil, Oman experienced rapid socio-economic growth and was ranked the fastest country to increase its Human Index Development from 1970 to 2010 [15]. The country’s Human Development Index continued to rise in this past decade, where it experienced a 17% increase from 2010 until 2020 [16]. In parallel with the Human Index Development improvement, obesity rates increased, while childhood mortality and malnutrition rates dropped [16,17,18]. To assess the nutrition status of community members, three national surveys were conducted in 1999, 2009, and 2017 [19,20,21]. The Oman National Nutrition Survey (ONNS), the most recent survey undertaken in 2017, assessed the prevalence and severity of malnutrition-related conditions among mothers and young children under five years of age. This paper builds on a recent publication that described the prevalence of malnutrition related disorders [21]. By employing the ONNS data, we aimed in this manuscript to assess feeding practices of children under two years of age and to correlate them with family-related parameters.

## 2. Materials and Methods

### 2.1. Survey and Sample Selection

The ONNS followed the WHO’s STEPwise approach to Surveillance, a comprehensive surveying tool to assemble, interpret, and disseminate data on NCDs [22]. The ONNS’s methodology was previously described [21,23]. Briefly, data collection was performed between December 2016 and April 2017. Households were selected based on a random selection from each census block by governorate, derived from the 2010 Oman census. The ONNS data was derived from households of Omani citizens only since data from the report was to be used to design the health development plan of the Ministry of Health. An a priori sample size of 375 households by governorate was deemed sufficient to power the national survey in answering the primary outcomes. As Muscat governorate hosts more non-Omani residents than other governorates, and since the original STEPS survey included Omani and non-Omani households, the number of households with Omani residents identified in Muscat’s governorate was inferior to the a priori sample size of 375 households. Accordingly, all households (n = 318) in Muscat’s governorate were selected in our analysis. Moreover, for this paper, inclusion criteria were for households that had at least one Omani citizen, had children under two years of age, and whose mothers were available in the household to answer the study questionnaires.

### 2.2. Tools and Definitions

Field workers were trained on data collection and performed field testing prior to studying household members. Interviews evaluated children’s BF practices, diet diversity, use of fortified foods, micronutrient supplementation, and the mothers’ educational level and marital status as well as the household income. A wealth index was evaluated using the principal component analysis method, incorporating indicators such as residence, water, sanitation, and possession of durable goods [14,15]. The wealth index was expressed as quintiles with the highest one referring to the wealthiest category and the lowest one to the poorest category.

Anthropometric measurements assessed children’s weight, length, and mid upper arm circumference, and the mothers’ weight, height, waist and hip circumferences [24].

Wasting and stunting were identified using weight-for-height z scores < −2 and height-for-age z score < −2.0, respectively. Children with a weight for length z score above 2.0 were identified as overweight/obese. Feeding practices were assessed using the WHO standards [25]. Early initiation of BF referred to lactation that was started within one hour of birth. Predominant BF identified infants who received breastmilk as the main source of feeding. Continued BF at 1 and 2 years referred to children who received breastmilk at 12–15 months and 20–23 months of age, respectively. Age appropriate BF assessed infants (0–5 months) who obtained breast milk exclusively, and 6–23 month-old children who received breast milk and complementary food in the previous day. Consumption of iron-fortified meals was evaluated in 6–23 month-old children who consumed an iron rich or fortified food in the previous day. Minimum Diet Diversity Score (DDS) assessed 6–23 month-old children who consumed from 4 or more groups. Children who met this score consumed at least one animal meal, one fruit or vegetable, and one staple food source in the day preceding the survey administration. Minimum Meal Frequency (MMF) assessed 6–8 months and 9–23 months breastfed children who received 2 and 3 meals daily, respectively. MMF identified 6–23 months, non-breastfed, children who were fed ≥4 times in the previous day. Minimum Acceptable Diet (MAD) identified 6–23 months old children who had the minimum DDS and the MFF [25]. Mothers were also asked about 0–6 month infants’ consumption of liquids such as water, infant formula, milk, juice drinks, soups, yoghurt, herbal infusions and teas.

### 2.3. Statistical Analysis

Categorical and continuous variables were presented using counts (percentages) and mean ± SD, respectively. Parametric tests were used for analysis in view of the large sample size and in line with the principles of the Central Limit Theorem [26]. Differences between variables were evaluated with the independent t-test for continuous variables, and the chi-square test for categorical variables. Univariate and multivariate logistic regression were performed for diet acceptability using the backward conditional method. Variables entered in the model were gender, child birthweight, child having stunting, wasting, overweight/obese category, BMI score, mother’s age, BMI, education, wealth quintile, and governorate. Variables that had *p* values < 0.15 at the univariate level were entered in the multivariate model. *p* values of <0.01 at the multivariate model were deemed significant. Missing variables were assessed using the WHO recommendations for handling missing data. We excluded cases that had missing values for a certain variable. We compared this method with replacing the missing cases with null figures [27]. Data analysis was conducted on IBM-SPSS (version 25.0; IBM Corp. Released 2017. IBM SPSS Statistics for Windows, IBM Corp., Armonk, NY, USA).

### 2.4. Ethical Considerations

ONNS’s research methodology followed the principles of the declaration of Helsinki, and approval for it was obtained from the Research and Ethical Review and Approval Committee at Oman’s Ministry of Health. After explaining to them the research methodology, mothers provided written informed consents to participate with their children in the survey. Participants were informed they were free to withdraw from the study at any point.

## 3. Results

Nutrition, lifestyle, and demographics information on 1344 pairs of mothers and children under two years of age enrolled in the ONSS were analyzed in this publication. Table 1 presents children and mothers’ characteristics. The majority of mothers had excess weight, a secondary level of education, and were not working outside their homes. Children had similar distribution across age and sex categories as well as wealth quintiles. Results revealed that 14, 10, and 6% of children had stunting, wasting, and excess weight, respectively. Al Wusta governorate had the highest representation across governorates and Muscat, Oman’s capital, the lowest (Table 1).

**Figure 1:**
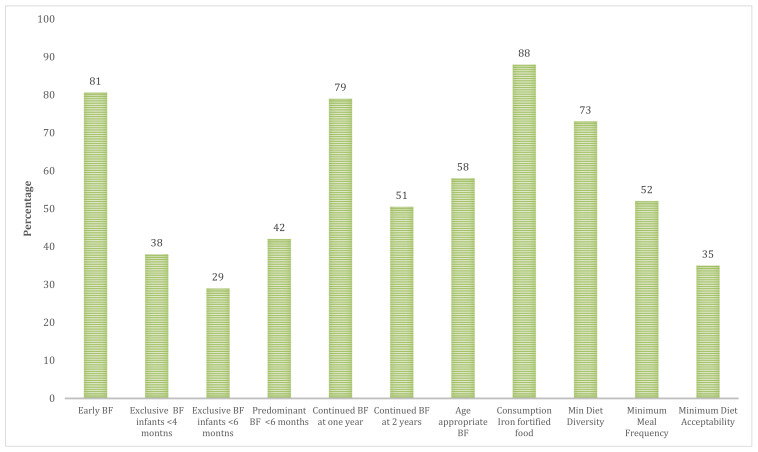
Children feeding indicators. BF: Breastfeeding.

**Figure 2:**
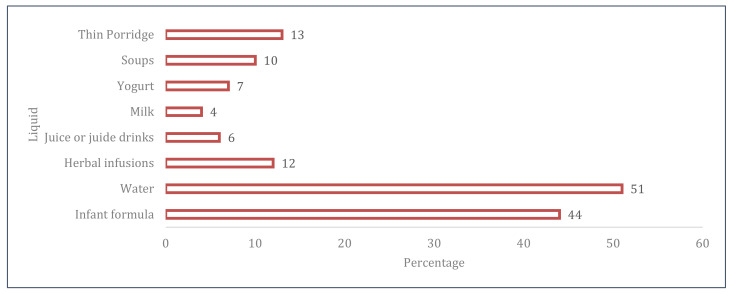
Liquids consumption among infants (0–6 months).

Figure 1 illustrates children BF and complementary feeding practices. Exclusive and predominant BF were provided to 29 and 42% of infants at six months, respectively. The majority (79%) and half (51%) of breastfed infants continued to receive breastmilk at one and two years, respectively. Age appropriate BF was noted in 58% of children under two years of age. Whereas 73% of children achieved the minimum DDS and 88% consumed an iron fortified meal in the previous day, only 35% of the sample met the criteria for minimum diet acceptability (Figure 1). Figure 2 presents the liquids that were consumed by infants under six months of age. Half of infants received water and infant formula milk. 

Moreover, 10–16% of infants received herbal infusions, thin porridge, or soups. A small percentage of infants consumed cow’s yogurt, milk, or juices (Figure 2).

Participants’ characteristics were tabulated by age appropriate BF and diet acceptability categories (Table 2 and Table 3). Mother education level, wealth quintile and governorate were significantly different across age-appropriate BF categories, with mothers with a secondary education and children having the lowest household wealth quintile being more likely to receive an age appropriate BF. Mothers with tertiary education were more likely to have children with age inappropriate BF. Differences were also noted among governorates with children from Al-Batinah North, Musandam, and Al-Wusta governorates most likely to receive BF appropriately (Table 2). In terms of diet acceptability, children with low birthweight and whose mothers were older, were more likely to have an acceptable diet. Children with non-acceptable diet were most likely to belong to the poorest households and to the governorates of Al Wusta and Dhofor (Table 3). Univariate and multivariate logistics regression of diet acceptability was tabulated in Table 4. Even though many variables were significant predictors of children’s diet acceptability at the univariate level, low birthweight and the governorate variable remained significant at the multivariate level, with children not having low birthweight and those from Al Wusta governorate having a higher likelihood of non-acceptable diet compared to residents of Muscat and those with low birthweight (Table 4).

## 4. Discussion

This publication provides insight into the feeding practices of children under two years of age and their association with mothers and household variables in the Sultanate of Oman. Results revealed that exclusive BF rates and MAD were low, whereas early BF, minimum diet diversity, and consumption of iron fortified foods were high. In addition to the governorate of assessed children, household wealth quintile, maternal education, and age also influenced age appropriate BF and/or diet acceptability.

Compared to the 2009 national survey, this study showed that exclusive BF at six months and age appropriate BF improved markedly from 16 to 29% and from 8 to 58%, respectively [20]. This improvement is likely a result of the educational campaigns and training that targeted women of reproductive age and health care providers, respectively, after the 2009 survey. Yet, rates of predominant BF at six months decreased from 94 to 42%, as well as those of MMF and MAD [20], while the proportion of children who continued to be breastfed at one at two years remain unchanged [20].

This study showed that Omani mothers head off to a good start with 84% providing breastmilk within an hour of birth, achieving the 2030 global target rate of BF of 70% set by the UNICEF and WHO [28] and exceeding the mean rate of middle and low income countries in the Middle East and Europe [29]. The success of early BF may be attributed to the availability of Baby Friendly hospitals across the country that were accredited as part of the global Breast Feeding Hospital Initiative (BFHI) launched in 1990 [30,31]. Yet, with a quarter of infants aged six months of age or less being breastfed exclusively, the Sultanate of Oman falls short of the BF target of 70% and the global average rate of 41% [28]. A recent cross sectional study in Oman revealed that around 25% of mothers provide infant formula within a week of birth, and our study showed that 44% of infants less than six months of age receive formula milk [32]. Surveys among mothers in Oman attributed reasons for early infant formula initiation to lack of self-efficacy of mothers, namely a perception that milk supply would be insufficient, and lack of assurance that babies would receive their nutritional needs from breastmilk while they are away [32,33]. Additional factors that negatively influence BF practices in the country include poor continuity of support, inadequate training of health care professionals as well as promotion of infant formula [30,31]. Even though all hospitals and facilities were originally accredited as part of the BFHI, many facilities in the country lost their accreditation of Baby Friendly, according to a recent analysis by the WHO that also showed poor implementation of BFHI [34].

Complementary feeding indicators such as MAD were better than worldwide figures [14,35] and similar to those of the United Arab Emirates, a high income country in the Gulf Cooperation Council and EMR [36]. Multivariate analysis of MAD revealed that governorate was the only significant determinant at the multivariate level, with Al Wusta governorate having the lowest diet acceptability in the country. Reasons may be attributed to the fact that this governorate is located in a desert area, with a predominant Bedouin population. Families usually reside far away from each other, and from health and food establishments. Unlike other governorates, Al Wusta has no dietitians from the Ministry of Health serving in its health care institutions, leading to suboptimal counseling and community awareness.

This study showed children coming from the poorest households were most likely to receive age appropriate BF, but had the lowest MAD proportions. This can be explained by the fact that breastmilk is more cost effective than infant formula and low Socio Economic Status (SES) may affect diet diversity by limiting access to nutritious food. BF rates were in line with those found in China, Indonesia, and Iran [37,38,39], but discord results from Australia and Sweden [40,41]. A similar association between diet diversity and SES was also observed in an analysis of global data [14]. Maternal education affected BF rates. Mothers with secondary education were more likely to provide age-appropriate BF compared to those with tertiary education. These findings are in line with results from China, but discord findings from Australia and Sweden [37,40,41]. Differences between countries is expected to be affected by factors such as interventions delivery, attendance of antenatal clinics, social welfare, and the support provided to families of young children [14].

Omani officials have been exploring strategies to improve BF and MAD rates. Recently, the Omani code that regulates the marketing of breast milk substitutes in line with the WHO and UNICEF guidelines was established [42,43,44]. The Ministry of Health in Oman in collaboration with the UNICEF also conducted a barrier analysis across several governorates in the country to assess determinants of exclusive BF and MAD, and to design strategies to enhance these outcomes. This study identified strategies to empower mothers to breastfeed, including the formation of support groups for new mothers as well as the recruitment of trainers who would develop educational material and train support group facilitators [33]. Similarly, this same study recommended strategies to enhance diet diversity and MAD among children under two. These initiatives included the formation of support groups for mothers of young children, training facilitators on proper means of education and coaching, and the recruitment of local supervisors and regional coordinators to ensure adequate implementation and follow up [33]. These initiatives are yet to be implemented in the country.

This study has several limitations and strengths. On the one hand, missing data is expected in such large national surveys. Rates were acceptable overall, except for the variable MMF, which was used to compute MAD, with 31% of the data missing. This was addressed using the WHO recommended methods for handling missing data and using the more conservative approach of excluding cases that had missing data. Another limitation was the lack of comprehensive assessment of fathers’ education level and nutritional determinants. On the other hand, this study has many strengths. It followed a robust methodology and comprehensively assessed young children as well as maternal socioeconomic and nutritional determinants. The study had a large sample size, providing adequate power of the assessed research questions, and adequately representing the population profile.

## 5. Conclusions

This study provides an update on Oman’s young children breastfeeding and complementary feeding practices from a national survey following the WHO STEPwise approach. Omani mothers were successful at initiating early BF after birth and at encouraging the consumption of iron rich meals. Yet, rates of exclusive BF rates and minimum diet acceptability were low. Compared to the last survey in 2009, age appropriate BF improved and minimum acceptable diet worsened. This study also highlighted the association between governorates, and household and maternal determinants with age appropriate BF and diet acceptability. Comprehensive strategies have been placed to influence the knowledge, attitude, and behavior of parents towards healthy feeding practices, and to provide the needed support to ensure the country progresses in reaching the goals for complementary and breast feeding practices for children under two. Future studies should evaluate the implementation of these initiatives in shaping feeding practices among young children in the country.

## Figures and Tables

**Table 1:** Mothers and children characteristics.

Characteristic		Result (n = 1344)
Child’s age (months), n (%)	0–5	343 (26)
	6–11	317 (24)
	12–17	355 (26)
	18–23	329 (24)
Child’s male sex, n (%)		673 (50)
Child with stunting, n (%)		178 (14)
Child with wasting, n (%)		118 (9.7)
Child with overweight or obesity, n (%)		73 (6.0)
Mother’s age (years), mean ± SD		31 ± 6.9
Mother’s BMI Category, n (%)	Underweight	46 (3.9)
	Normal Weight	327 (28)
	Overweight or obese	804 (68)
Mother’s education Level, n (%)	Less than Primary	80 (6.3)
	Primary	59 (4.7)
	Secondary	763 (60)
	Tertiary	362 (29)
Mother working outside her home, n (%)		188 (14)
Wealth quintile, n (%)	Poorest	225 (17)
	Poor	270 (20)
	Middle	279 (21)
	Wealthy	266 (20)
	Wealthiest	284 (21)
Governorate, n (%)	Muscat	74 (5.5)
	Dhofar	148 (11)
	Al-Dakhlya	143 (11)
	Al-Sharqyah North	102 (7.6)
	Al-Sharqyah South	112 (8.3)
	Al-Batinah North	112 (8.3)
	Al-Batinah South	116 (8.6)
	Al-Dhahairah	147 (11)
	Al-Buraimy	129 (10)
	Musandam	98 (7.3)
	Al-Wusta	163 (12)

BMI: Body Mass Index, Categorical and continuous variables are presented using counts (percentages) and mean ± SD, respectively.

**Table 2:** Participants’ characteristics by age appropriate BF categories.

Variable		Age Appropriate BF (n = 708)	Age Inappropriate BF (n = 508)	*p* Value
Mothers’ BMI (Kg/m^2^), mean ± SD		29 ± 6.3	28 ± 7.0	0.674
Mother’s age (years), mean ± SD		31 ± 7.0	31 ± 6.9	0.292
Mother education level, n (%)	Less than Primary	43 (6)	31 (7)	0.015
	Primary	36 (5)	21 (4)	
	Secondary	430 (63) ^B^	260 (55)	
	Tertiary	169 (25)	157 (33) ^A^	
Mother working outside her home, n (%)		98 (14)	69 (14)	0.897
Child’s Sex, n (%)	Male	353 (50)	258 (51)	0.749
	Female	355 (50)	250 (49)	
Birthweight < 2500 g, n (%)		78 (11)	70 (14)	0.132
Child with stunting, n (%)		81 (14)	37 (13)	0.727
Child with wasting, n (%)		60 (11)	18 (6.4)	0.050
Child with overweight or obesity, n (%)		20 (3.5)	13 (4.6)	0.427
Child’s BMI z score (standardized for age and sex)		−0.26 ± 1.5	−0.23 ± 1.4	0.804
Wealth quintile, n (%)	Poorest	138 (20) ^B^	66 (13)	0.020
	Poor	149 (21)	99 (20)	
	Middle	149 (21)	105 (21)	
	Wealthy	127 (18)	112 (23)	
	Wealthiest	139 (20)	115 (23)	
Governorate, n (%)	Muscat	37 (5)	31 (6)	<0.01
	Dhofor	62 (9)	64 (13) ^A^	
	Al Dhakhlya	57 (8)	67 (13) ^A^	
	Al-Sharqyah North	58 (8)	32 (6)	
	Al-Sharqyah South	54 (8)	45 (9)	
	Al-Batinah North	72 (10) ^B^	35 (7)	
	Al-Batinah South	67 (9)	44 (9)	
	Al-Dhahairah	66 (9)	68 (13) ^A^	
	Al-Buraimy	63 (9)	55 (11)	
	Musandam	64 (9) ^B^	26 (5)	
	Al-Wusta	108 (15) ^B^	41 (8)	

BMI: Body Mass Index; ^A^ and ^B^ reflect statistically significant differences across columns. Categorical and continuous variables are presented using counts (percentages) and mean ± SD, respectively.

**Table 3:** Participants’ characteristics by acceptable diet categories.

Variable		Acceptable Diet (n = 277)	Non Acceptable Diet (n = 508)	*p* Value
Sex, n (%)	Male	142 (51)	251 (49)	0.620
	Female	135 (49)	257 (51)	
Birthweight < 2500 g, n (%)		26 (10)	72 (15) ^A^	0.039
Child with stunting, n (%)		39 (15)	61 (13)	0.412
Child with wasting, n (%)		27 (11)	41 (8.8)	0.412
Child with overweight or obesty, n (%)		9 (3.5)	15 (3.2)	0.809
Child’s BMI z score (standardized for age and sex)		−0.32 ± 1.4 ^A^	−0.20 ± 1.3	0.279
Mothers’ BMI (Kg/m^2^), mean ± SD		28 ± 6.4	29 ± 6.6 ^A^	0.112
Mother’s age (years), mean ± SD		33 ± 6.0 ^A^	31 ± 7.1	<0.01
Mother education level, n (%)	Less than Primary	6 (2.3)	43 (9) ^A^	0.04
	Primary	13 (4.9)	27 (5.7)	
	Secondary	173 (65)	287 (60)	
	Tertiary	74 (28)	120 (25)	
Mother working outside her home, n (%)		37 (13)	68 (13)	0.897
Wealth quintile, n (%)	Poorest	27 (9.8)	123 (25) ^A^	<0.01
	Poor	73 (27) ^B^	91 (18)	
	Middle	56 (20)	97 (19)	
	Wealthy	59 (21)	93 (19)	
	Wealthiest	60 (22)	97 (19)	
Governorate, n (%)	Muscat	23 (8.3) ^B^	14 (2.8)	<0.01
	Dhofor	18 (6.5)	69 (14) ^A^	
	Al Dhakhlya	34 (12) ^B^	34 (6.7)	
	Al-Sharqyah North	37 (13) ^B^	20 (3.9)	
	Al-Sharqyah South	17 (6.1)	49 (9.6)	
	Al-Batinah North	43 (16) ^B^	32 (6.3)	
	Al-Batinah South	32 (12) ^B^	36 (7.1)	
	Al-Dhahairah	23 (8.3)	63 (12)	
	Al-Buraimy	31 (11)	43 (8.5)	
	Musandam	15 (5.4)	45 (8.9)	
	Al-Wusta	4 (1.4)	103 (20) ^A^	

BMI: Body Mass Index; ^A^ and ^B^ reflect statistically significant differences across columns. Categorical and continuous variables are presented using n (%) and mean ± SD, respectively.

**Table 4:** Logistic Regression of acceptable diet by selected variables.

Variable	Univariate Analysis			Multivariate Analysis		
	OR	95% CI	*p* Value	AOR	95% CI	*p* Value
Child’s Female sex	1.08	0.80–1.4	0.620			
Child’s Birthweight > 2500 g	0.609	0.38–0.98	0.041	0.541	0.31–0.94	0.029
Child with stunting, n (%)	1.12	0.78–1.85	0.412			
Child with wasting, n (%)	1.24	0.74–2.07	0.412			
Child with overweight or obesity, n (%)	1.11	0.48–2.57	0.809			
Child’s BMI z score	1.06	0.95–1.2	0.279			
Mother’s age (years)	0.962	0.94–0.99	<0.01	0.985	0.96–1.0	0.296
Mothers’ BMI (Kg/m^2^)	1.02	1.0–1.05	0.112	1.01	0.98–1.0	0.700
**Mother’s education Level**			0.010			0.418
Less than primary (referent)	1	-	-	1	-	-
Primary	0.290	0.01–0.85	0.025	0.946	0.25–3.6	0.936
Secondary	0.231	0.097–0.56	<0.01	0.565	0.19–1.7	0.302
Tertiary	0.226	0.092–0.59	<0.01	0.700	0.22–2.2	0.541
Mother working outside her home	1.0	0.65–1.5	0.991			
**Wealth Quintile**			<0.01			0.125
Poorest (referent)	1	-	-	1	-	-
Poor	0.27	0.16–0.46	<0.01	0.558	0.28–1.1	0.090
Middle	0.38	0.22–0.65	<0.01	0.900	0.46–1.8	0.819
Wealthy	0.35	0.20–0.59	<0.01	1.10	0.54–2.2	0.797
Wealthiest	0.36	0.21–0.60	<0.01	0.90	0.45–1.9	0.836
**Governorate**			<0.01			<0.01
Muscat (referent)	1	-	-	1	-	-
Dhofor	6.3	2.7–15	<0.01	5.6	2.3–14	<0.01
Al Dhakhlya	1.64	0.73–3.7	0.23	1.7	0.73–4.2	0.207
Al-Sharqyah North	0.89	0.38–2.1	0.79	0.8	0.32–2.0	0.636
Al-Sharqyah South	4.74	1.99–11	<0.01	5.7	2.2–15	<0.01
Al-Batinah North	1.22	0.55–2.7	0.63	1.2	0.51–3.0	0.640
Al-Batinah South	1.85	0.82–4.2	0.14	2.2	0.92–5.2	0.078
Al-Dhahairah	4.50	2.0–10	<0.01	3.3	1.4–8.1	<0.01
Al-Buraimy	2.28	1.0–5.1	0.05	2.5	1.0–6.2	0.040
Musandam	4.93	2.0–12	<0.01	5.9	2.3–15	<0.01
Al-Wusta	42.30	13–140	<0.01	34.6	10–119	<0.01

Variables that had a *p* value < 0.15 in the univariate regression were incorporated in the multivariate analysis model. OR: Odds Ratio; AOR: Adjusted Odds Ratio, 95% CI: 95% confidence interval; BMI: Body Mass Index. n (%) reflect the counts (percentages).

## Data Availability

Third party data restrictions apply to the availability of these data. Data was obtained from the Ministry of Health, from the authors Salima Al Mamari and Saleh Al Shammkhi.

## References

[1] Robertson R.C., Manges A.R., Finlay B.B., Prendergast A.J. (2019). The Human Microbiome and Child Growth—First 1000 Days and Beyond. Trends Microbiol..

[2] Schwarzenberg S.J., Georgieff M.K. (2018). Advocacy for Improving Nutrition in the First 1000 Days to Support Childhood Development and Adult Health. Pediatrics.

[3] Walters D.D., Phan L.T.H., Mathisen R. (2019). The cost of not breastfeeding: Global results from a new tool. Health Policy Plan..

[4] Owais A., Schwartz B., Kleinbaum D.G., Suchdev P.S., Faruque A., Das S.K., Stein A.D. (2016). Minimum acceptable diet at 9 months but not exclusive breastfeeding at 3 months or timely complementary feeding initiation is predictive of infant growth in rural Bangladesh. PLoS ONE.

[5] Mallard S.R., Houghton L.A., Filteau S., Mullen A., Nieuwelink J., Chisenga M., Siame J., Gibson R.S. (2014). Dietary Diversity at 6 Months of Age Is Associated with Subsequent Growth and Mediates the Effect of Maternal Education on Infant Growth in Urban Zambia. J. Nutr..

[6] Marriott B.P., White A., Hadden L., Davies J.C., Wallingford J.C. (2012). World Health Organization (WHO) infant and young child feeding indicators: Associations with growth measures in 14 low-income countries. Matern. Child Nutr..

[7] Krasevec J., An X., Kumapley R., Bégin F., Frongillo E.A. (2017). Diet quality and risk of stunting among infants and young children in low-and middle-income countries. Matern. Child Nutr..

[8] UNICEF, WHO (2021). The Extension of the 2025 Maternal, Infant and Young Child Nutrition Targets to 2030.

[9] World Health Organization (2009). Infant and Young Child Feeding Model Chapter for Textbooks for Medical Students and Allied Health Professionals.

[10] WHO Infant and Young Child Feeding. https://www.who.int/data/nutrition/nlis/info/infant-and-young-child-feeding.

[11] Rito A.I., Buoncristiano M., Spinelli A., Salanave B., Kunešová M., Hejgaard T., García Solano M., Fijałkowska A., Sturua L., Hyska J. (2019). Association between Characteristics at Birth, Breastfeeding and Obesity in 22 Countries: The WHO European Childhood Obesity Surveillance Initiative—COSI 2015/2017. Obes. Facts.

[12] The Lancet (2016). Breastfeeding: Achieving the new normal. Lancet.

[13] Chowdhury R., Sinha B., Sankar M.J., Taneja S., Bhandari N., Rollins N., Bahl R., Martines J. (2015). Breastfeeding and maternal health outcomes: A systematic review and meta-analysis. Acta Paediatr..

[14] White J.M., Bégin F., Kumapley R., Murray C., Krasevec J. (2017). Complementary feeding practices: Current global and regional estimates. Matern. Child Nutr..

[15] UNDP (2010). The Real Wealth of Nations: Pathways to Human Development. Human Development Report 2010.

[16] UNDP (2020). The Next Frontier: Human Development and the Anthropocene. Human Development Report 2010.

[17] World Obesity Federation Oman. https://data.worldobesity.org/country/oman-165/#data_trends.

[18] Ng M., Fleming T., Robinson M., Thomson B., Graetz N., Margono C., Mullany E.C., Biryukov S., Abbafati C., Abera S.F. (2014). Global, regional, and national prevalence of overweight and obesity in children and adults during 1980–2013: A systematic analysis for the Global Burden of Disease Study 2013. Lancet.

[19] Alasfoor D., Elsayed M., Al Qasmi A., Malankar P., Sheth M., Prakash N. (2007). Protein-energy malnutrition among preschool children in Oman: Results of a national survey. EMHJ—East. Mediterr. Health J..

[20] Elsayed M., Al-Shammkhi S. (2009). Second National Health Survey for Protein Energy Malnutrition in Children below Five Years of Age in the Sultanate of Oman: 2008–2009 Analysis Report.

[21] Petry N., Al-Maamary S.A., Woodruff B.A., Alghannami S., Al-Shammakhi S.M., Al-Ghammari I.K., Tyler V., Rohner F., Wirth J.P. (2020). National Prevalence of Micronutrient Deficiencies, Anaemia, Genetic Blood Disorders and Over-and Undernutrition in Omani Women of Reproductive Age and Preschool Children. Sultan Qaboos Univ. Med. J..

[22] WHO (2017). The WHO STEPwise Approach to Noncommunicable Disease Risk Factor Surveillance.

[23] Almaamary S., Al Shammakhi S., Alghamari I., Jabbour J., Al-Jawaldeh A. (2021). Preschoolers’ and Mothers Dietary Practices and Compliance with the 24-h Movement Guidelines: Results of Oman’s National Nutrition Survey. Int. J. Environ. Res. Public Health.

[24] WHO (2008). WHO STEPwise Approach to Surveillance.

[25] World Health Organization (2008). Indicators for Assessing Infant and Young Child Feeding Practices: Part 1: Definitions: Conclusions of a Consensus Meeting Held 6-8 November 2007 in Washington DC, USA.

[26] Ghasemi A., Zahediasl S. (2012). Normality tests for statistical analysis: A guide for non-statisticians. Int. J. Endocrinol. Metab..

[27] WHO (2010). Indicators for Assessing Infant and Young Child Feeding Practices: Part 2 Measurement.

[28] Collective G.B. (2018). Global Breastfeeding Scorecard, 2018. Enabling Women to Breastfeed through Better Policies and Programmes.

[29] Oakley L., Benova L., Macleod D., Lynch C.A., Campbell O.M. (2018). Early breastfeeding practices: Descriptive analysis of recent demographic and health surveys. Matern. Child Nutr..

[30] Al-Nuaimi N., Katende G., Arulappan J. (2017). Breastfeeding Trends and Determinants: Implications and recommendations for Gulf Cooperation Council countries. Sultan Qaboos Univ. Med. J..

[31] Sinani M.A. (2008). Breastfeeding in Oman-The way forward. Oman Med. J..

[32] Al-Barwani S. (2017). Employing the Theory of Planned Behavior to Predict Breastfeeding Intention and Intensity in Oman.

[33] Ministry of Health (2019). Barrier Analysis of Exclusive Breastfeeding, Feeding Frequency among Children, and Iron Supplementation among Pregnant Women in the Sultanate of Oman.

[34] WHO (2017). National Implementation of the Baby-Friendly Hospital Initiative 2017.

[35] Onyango A.W., Borghi E., de Onis M., del Carmen Casanovas M., Garza C. (2014). Complementary feeding and attained linear growth among 6–23-month-old children. Public Health Nutr..

[36] Taha Z., Garemo M., Nanda J. (2020). Complementary feeding practices among infants and young children in Abu Dhabi, United Arab Emirates. BMC Public Health.

[37] Chen C., Cheng G., Pan J. (2019). Socioeconomic status and breastfeeding in China: An analysis of data from a longitudinal nationwide household survey. BMC Pediatr..

[38] Ajami M., Abdollahi M., Salehi F., Oldewage-Theron W., Jamshidi-Naeini Y. (2018). The Association between Household Socioeconomic Status, Breastfeeding, and Infants’ Anthropometric Indices. Int. J. Prev. Med..

[39] Sebayang S.K., Dibley M.J., Astutik E., Efendi F., Kelly P.J., Li M. (2020). Determinants of age-appropriate breastfeeding, dietary diversity, and consumption of animal source foods among Indonesian children. Matern. Child Nutr..

[40] Flacking R., Nyqvist K.H., Ewald U. (2007). Effects of socioeconomic status on breastfeeding duration in mothers of preterm and term infants. Eur. J. Public Health.

[41] Amir L.H., Donath S.M. (2008). Socioeconomic status and rates of breastfeeding in Australia: Evidence from three recent national health surveys. Med. J. Aust..

[42] Ministry of Health (2014). National Nutrition Strategy 2014–2050.

[43] WHO (1981). International Code of Marketing of Breast-Milk Substitutes.

[44] Ministry of Health The Omani Code for Marketing of Breast Milk Substitutes Reviewed. https://www.moh.gov.om/en/-/--1092.

